# Effects of Special Therapeutic Footwear on the Prevention of Diabetic Foot Ulcers: A Systematic Review and Meta-Analysis of Randomized Controlled Trials

**DOI:** 10.1155/2022/9742665

**Published:** 2022-09-26

**Authors:** Bo Luo, Yuying Cai, Dawei Chen, Chun Wang, Hui Huang, Lihong Chen, Yun Gao, Xingwu Ran

**Affiliations:** ^1^Innovation Center for Wound Repair, Diabetic Foot Care Center, Department of Endocrinology and Metabolism, West China Hospital, Sichuan University, Chengdu, 610041 Sichuan, China; ^2^West China Medical School, Sichuan University, Chengdu, 610041 Sichuan, China

## Abstract

**Objective:**

To reduce diabetic foot ulcer (DFU) occurrence or recurrence, diabetic therapeutic footwear is widely recommended in clinical practice for at-risk patients. However, the effectiveness of therapeutic footwear is controversial. Thus, we performed a systematic review and meta-analysis of randomized controlled trials (RCTs) to examine whether special therapeutic footwear could reduce the incidence of DFU.

**Method:**

We systematically searched multiple electronic databases (Medline, EMBASE, and EMB databases) to identify eligible studies published from inception to June 11, 2021. The database search, quality assessment, and data extraction were independently performed by two reviewers. Efficacy (i.e., incidence of DFU) was explored using the R'meta' package (version 4.15-1). To obtain more robust results, the random-effects model and the Hartung-Knapp-Sidik-Jonkman method were selected to assess pooled data. Metaregression analysis and sensitivity analysis were performed to explore heterogeneity, and publication bias was assessed by a visual inspection of funnel plots and the AS-Thompson test.

**Results:**

Eight RCTs with a total of 1,587 participants were identified from the search strategy. Compared with conventional footwear, special therapeutic footwear significantly reduced the incidence of DFU (RR 0.49; 95% CI, 0.28-0.84), with no evidence of publication bias (*P* = 0.69). Unexpectedly, the effectiveness of special therapeutic footwear had a reverse correlation with the intervention time (coefficient = 0.085, *P* < 0.05) in the metaregression analysis.

**Conclusion:**

Special therapeutic footwear with offloading properties is effective in reducing the incidence of DFU. However, the effect may decrease gradually over time. Despite undefined reasons, the optimal utility time and renewal frequency of special therapeutic footwear should be considered.

## 1. Introduction

Diabetic foot ulcer (DFU), a major complication of diabetes mellitus (DM), is not uncommon and is linked to high-normal levels of morbidity and mortality as well as enormous economic costs. The lifetime risk for the development of a foot ulcer in a patient with DM is estimated to be 19-34% [1]. Diabetes-related foot ulcers precede at least 60% of all nontraumatic lower limb amputations [2]. Moreover, even after the resolution of a foot ulcer, recurrence is also common [1]. The annual incidence of DFU increases by 31.6% in the presence of a history of foot ulceration [3]. Therefore, the prevention of ulcer occurrence or recurrence is of prime importance in the current approach to DFU.

Abnormal biomechanical stress, including elevated vertical pressure and horizontal shear pressure, accounts for the development of a foot ulcer, especially acting on the foot during ambulation. High levels of mechanical pressure contribute to approximately 50% of DFUs during repetitive weight-bearing activity [1, 4–6]. Thus, foot ulceration is probably the most preventable of all the complications of diabetes [7]. Offloading, namely, reducing supranormal mechanical pressure, is considered the cornerstone of preventing foot ulcer occurrence or recurrence [8–12].

To prevent diabetic foot ulcers, various offloading interventions (e.g., offloading devices, special therapeutic footwear, surgery, and other offloading interventions) are utilized in clinical practice worldwide [11–16]. Among these offloading methods, special therapeutic footwear, recommended by the International Working Group on the Diabetic Foot (IWGDF) for persons at risk for foot ulceration (IWGDF risk 2-3) [8], was demonstrated to be capable of redistributing the pathological mechanical pressure and relieving the abnormal load on the plantar foot surface (i.e., the weight-bearing surface of the foot) [8, 17–19] and could be routinely worn at all times, both indoors and outdoors [8].

Unfortunately, few studies provide strong evidence on the efficacy of special therapeutic footwear. Thus, the quality of evidence for the recommendation of special therapeutic footwear to prevent DFU remains low [8]. Therefore, the aims of this paper were to systematically review published randomized controlled trials (RCTs) and conduct a comprehensive meta-analysis to evaluate the efficacy of reducing foot ulcer occurrence or recurrence in the presence of special therapeutic footwear to provide powerful evidence supporting the rational prescription of special therapeutic footwear in clinical practice.

## 2. Materials and Methods

The systematic search was performed according to the preferred reporting items for systematic reviews and meta-analyses (PRISMA) [20].

### 2.1. Search Strategy

Two authors (BL and YYC), trained in health research methods, performed a systematic literature search of Medline via OVID, Embase via OVID, and all EBM databases via OVID from inception to June 11, 2021. MeSH combined with free word terms about “Diabetic Foot”, “Foot Ulcer”, “walking”, “walkers”, “shoe”, and “orthotic Devices” were used to identify relevant articles. We also screened the reference lists of published reviews to identify additional relevant studies. A full overview of the specific searches per database is provided in Appendix 1.

### 2.2. Inclusion and Exclusion Criteria

We included randomized controlled trials (RCTs) that compared special therapeutic footwear against conventional footwear in an at-risk adult population with DM. The special therapeutic footwear, including extra-depth shoes, custom-made shoes, custom-made insoles, or toe orthoses, was defined based on IWGDF guidelines on the prevention and management of diabetic foot disease [8]. Exclusion criteria included (1) all case reports, case series, cross-sectional, letters to the editor, opinion pieces, conference proceedings, and editorials and animal studies, (2) patients with Charcot foot or patients with current (active or unhealed) foot ulceration and requiring treatment, and (3) combined offloading measures as intervention.

If multiple published reports from the same study were available, we included only the one with the most detailed information for both intervention and outcome. No language restriction was applied.

### 2.3. Study Screening and Data Extraction

After the removal of duplicates, two authors (BL and YYC) independently screened the titles/abstracts to identify all potentially eligible articles. Both authors then read the full texts of these articles and discussed the final list of included articles to reach consensus. Any discrepancy was resolved in consultation with a third review (YG). Data were extracted by one author (BL) and supervised by a second author (YYC). The primary extracted data included (1) authors; (2) year of publication; (3) study design; (4) sample size; (5) length of follow-up; (6) follow-up rate; (7) sex, age, body mass index (BMI), glycosylated hemoglobin (HbA1c), and duration of diabetes; and (8) the intervention and outcomes of interest. In the present study, the main outcome of interest was the risk of DFU.

### 2.4. Risk of Bias Assessment

The methodological quality of the included studies was assessed with a modified version of the Cochrane Collaboration tool [21]. This tool was designed to evaluate the risk of bias for randomized studies and includes six domains: randomization, blinding, allocation concealment, incomplete outcome data, selective outcome reporting, and sample size estimate.

The quality of evidence was evaluated using the GRADE (Grading of Recommendations Assessment, Development, and Evaluation) working group classification [22, 23]. The GRADE approach categorized evidence from the included studies into high, moderate, low, or very low quality.

### 2.5. Statistical Analysis

The meta-analysis was conducted using R' (version 4.0.3), meta' package (version 4.15-1), metafor' package (version 2.4-0), and dmetar' package (https://dmetar.protectlab.org/). The results are presented with 95% confidence intervals (CIs). Estimates for dichotomous outcomes (e.g., foot ulceration: yes or no) were reported as relative risk (RR). The overall relative risk (RR) and 95% CI were calculated by pooling RRs between the intervention group and the control group provided by the original studies using a random-effect model. The Hartung-Knapp-Sidik-Jonkman method was performed to reduce type I error [24].

Statistical heterogeneity between studies was measured using the *Q*-statistic, Tau^2^-statistic, *H*-statistic, and *I*^2^-statistic. *I*^2^ was interpreted based on a “rule of thumb” (*I*^2^ = 25%: low heterogeneity; *I*^2^ = 50%: moderate heterogeneity; *I*^2^ = 75%: substantial heterogeneity) [24]. Between-study heterogeneity was explored by searching for outliers. A study was defined as an outlier when its effect size estimate was so extreme that we have high certainty that the study cannot be part of the “population” of effect sizes we actually pooled in our meta-analysis (i.e., the individual study differs significantly from the overall effect). Additionally, to assess whether studies might exert a very high influence on our overall results and then distort our pooled effect, an influence analysis was performed using the leave-one-out method.

A metaregression analysis was performed to explore the possible source of heterogeneity. At the beginning of the metaregression analysis, multimodel inference was used to comprehensively identify possible predictor combinations that provided the best fit for the metaregression model, and the mixed-effects model was finally employed in the metaregression analysis. Before reporting the results, we tested the robustness of the metaregression model using a permutation test [25].

Publication bias was detected by visually examining the symmetry of the funnel plot and the AS-Thompson test [26].

## 3. Results

### 3.1. Search Results

**Figure 1:**
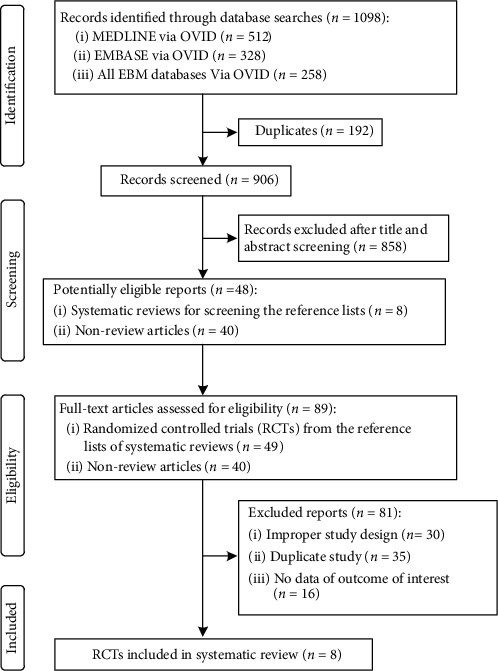
Flow chart for identifying eligible studies.

As shown in Figures 1, 906 records were retrieved by the literature search. After study assessment, we identified 8 RCTs [6, 27–33] that met our inclusion criteria.

### 3.2. Characteristics of Included Studies

The characteristics of the 8 included trials with 1587 participants are summarized in Table 1. Overall, the included studies were conducted in 4 different countries: 3 in Italy, 3 in the USA, 1 in Brazil, and 1 in the Netherlands. These studies enrolled 53–400 patients (mean age range of 56–70, mean baseline HbA1c range of 7.6–8.7%, and mean duration of diabetes range of 12 to 18 years). The duration of follow-up ranged from 3 to 24 months. Of the included studies, a total of 923 (58.2%) participants had a history of foot ulcers. Based on the Risk Classification System of IWGDF [8], more than 96% of the included patients had a moderate or high ulcer risk (IWGDF risk 2-3).

### 3.3. Risk of Bias Assessment

Table 2 summarizes the methodological quality of the included studies. Of the 8 RCTs, 6 (75%) [6, 28–30, 32, 33] reported adequate random sequence generation, and 2 (25%) [27, 31] were probably adequately generated random sequences; 2 (25%) [6, 32] definitely blinded patients and 5 (62.5%) [6, 27, 28, 30, 33] definitely blinded outcome assessors. Three RCTs (37.5%) [27, 28, 33] definitely conducted sample size estimates. All 8 RCTs (100%) reported complete outcome data and were free from selective reporting.

### 3.4. Special Therapeutic Footwear and the Incidence of Foot Ulcers

**Figure 2:**
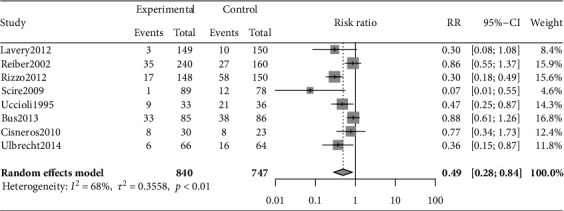
Forest plot of the effect of special therapeutic footwear in reducing the incidence of diabetes-related foot ulcers in 8 RCT studies including 1,587 participants and 302 events. Results are expressed as relative risk (RR) and 95% confidence intervals (95% CI). Pooled analysis *P* < 0.05; heterogeneity test: *I*^2^ = 68%, *P* < 0.01.

The incidence of DFU was reported in all 8 RCTs [6, 27–33]. Compared with conventional footwear, special therapeutic footwear significantly reduced foot reulceration or ulceration (RR 0.49, 95% CI, 0.28 to 0.84; Figure 2). Moderate heterogeneity existed in the overall analysis (*I*^2^ = 68%, *P* < 0.01).

### 3.5. The Efficacy of Special Therapeutic Footwear and Intervention Time (Duration of Follow-Up)

**Figure 3:**
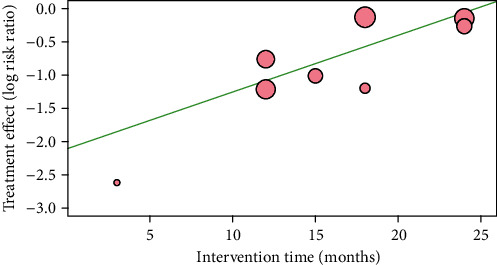
Metaregression analysis of the association between the efficacy of special therapeutic footwear and intervention time.

In the metaregression analysis, which was used to explore the possible source of heterogeneity, the multimodel influence showed that intervention time as the predictor was the best fitting model for further analysis. Subsequently, the metaregression model with intervention time as the predictor indicated that the effect of special therapeutic footwear gradually decreased as the intervention time period was extended (coefficient = 0.085, *P* = 0.015; Figure 3).

### 3.6. Publication Bias

**Figure 4:**
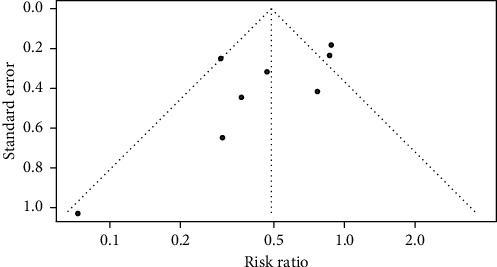
Funnel plot for publication bias.

As shown in Figure 4, an asymmetric funnel plot suggested possible publication bias. To further define whether publication bias existed, the AS-Thompson test was performed. However, the AS-Thompson test did not show statistical significance (*P* = 0.69), which suggested that no evidence of publication bias existed.

## 4. Discussion

In this study, we extracted data from all RCTs published in the field of special therapeutic footwear to comprehensively evaluate their effectiveness in preventing foot ulcers in populations with diabetes. The results demonstrated that special therapeutic footwear provided a clear benefit in preventing ulcer occurrence or recurrence compared with conventional footwear.

Unlike previous systematic reviews, the present study only included RCTs comparing special therapeutic footwear and conventional footwear, which could provide consistent outcomes to explore the overall effect and obtain high-quality evidence. In a recent systematic review, Ahmed et al. summarized and evaluated the evidence for footwear and insole features for reducing the occurrence of diabetic neuropathy ulceration. However, this review was only a descriptive summary of outcome measures from twenty-five studies with five different study designs, instead of combining results in a statistically sound manner. Similarly, the other five earlier systematic reviews were also limited to conducting a structured literature review [11, 34–37]. Due to the different study designs and diverse results of the included studies, their structured literature review did not yield consistent and strong evidence to support the clinical benefits of special therapeutic footwear in preventing foot ulcer occurrence. In contrast, the present meta-analysis employed rigorous statistical methods to merge consistent outcomes from the included RCTs and then yielded a robust conclusion.

Interestingly, we observed in the metaregression analysis that the protective effect of special therapeutic footwear gradually decreased as the intervention time increased. This finding suggests that the efficacy of specialized therapeutic footwear in preventing foot ulcers might diminish over time, which was rarely noticed in previous relevant studies. The potential mechanisms underlying this finding are not fully understood. Causative mechanisms may include the following: (1) the gradual declining compliance of the patients may be responsible. Several studies have suggested that adherence to wearing special therapeutic footwear is paramount for the effectiveness of preventing foot ulcers [6, 18, 19]. Regrettably, few studies have explored the association between intervention time and adherence. In a small-sample study of the Dutch population, Keukenkamp et al. explored the effect of using motivational interviewing to improve footwear adherence in individuals with diabetes who were at high risk for foot ulceration and had low footwear adherence. This study showed that median footwear adherence at home was 67% at baseline, 90% at one week, and 56% at 3 months in the motivational interviewing group and 35%, 33%, and 31%, respectively, in the standard education group. These data indirectly indicated that footwear adherence was inclined to worsen with increasing intervention time, despite the intensity of education activities [38]. However, studies that can provide direct evidence are needed to confirm this correlation and identify potential causes in the future. (2) Alternatively, the wear and aging of special therapeutic footwear during intervention may be responsible. Empiric evidence supports the important role of the ruggedness of special therapeutic footwear in the effectiveness of preventing foot ulcers. However, the correlation between these factors has not yet been explored due to the considerable differences in footwear materials, design features, and patients' habits of walking and usage. Therefore, the optimal utility time and renewal frequency for one pair of special therapeutic footwear have not yet been established. More related RCTs or observational studies should focus on the correlation between the ruggedness of special therapeutic footwear and the effectiveness of preventing foot ulcers in the future.

A recent meta-analysis partially explored individuals who are most likely to benefit. In this study, Crawford et al. performed a subgroup analysis based on whether the subjects of the included trials had a history of foot ulceration. In the subgroup with a history of foot ulcers, special therapeutic footwear did not significantly reduce foot ulcer occurrence (RR 0.71; 95% CI, 0.47-1.06). However, opposite results were observed in the subgroup without a history of foot ulcers (data not shown). They concluded that special therapeutic footwear might be more beneficial to patients without a history of foot ulcers [39]. However, when the subjects included in our meta-analysis were stratified according to the presence or absence of healed DFUs, we did not find a significant correlation between the effectiveness of special therapeutic footwear and a history of DFUs in the metaregression analysis (*P* = 0.64). In other words, the patients who had no history of DFU did not receive a greater benefit from special therapeutic footwear than those who had a history of DFU. We also performed a subgroup analysis in four RCTs [6, 28, 31, 33] in which all participants had a history of DFU. The results showed that special footwear tended to decrease the risk of foot ulcer recurrence, but this correlation did not reach statistical significance (RR 0.66 [95% CI, 0.34-1.28], *P* = 0.140) (shown in Appendix 3). This controversy might be attributed to the fact that the performance of subgroup analysis under the condition of a limited number of included studies may lead to unstable results. Thus, more carefully designed and adequately powered studies (both RCTs and observational studies) are warranted to examine whether the effect of special therapeutic footwear differs among patients with or without a history of DFU. From a physiopathological point of view, elevated mechanical stress in the presence of a loss of protective sensation (LOPS) is one of the most common causes of DFU [1]. Peripheral neuropathy can also cause further changes in gait, foot deformity, and soft tissue, all of which can further increase mechanical stress [40]. Thus, the combination of LOPS and elevated mechanical stress leads to tissue damage and DFU [1, 13]. The use of special therapeutic footwear is only intended to help relieve excessive mechanical stress at the plantar and dorsal surfaces of the foot. As foot deformity is one of the common reasons for increased mechanical stress [8], patients with LOPS+foot deformity should benefit more from the use of special therapeutic footwear. For patients with peripheral artery disease (PAD), the severity of PAD may influence the benefits. In patients with severe PAD (e.g., interstitial claudication or rest pain), the main reason for foot ulcers may be tissue ischemia and dysfunction instead of increased mechanical stress [41]. Thus, patients with severe PAD may have fewer benefits from the use of special therapeutic footwear. However, if PAD is mild and does not severely impair blood supply to the feet, patients with mild PAD+foot deformity may also benefit more from the use of special therapeutic footwear. Additionally, most patients with a history of DFU often have elevated mechanical stress at the plantar and dorsal surfaces of the foot, and patients with a minor lower-extremity amputation usually develop foot deformities [42]. Thus, among patients with an IWGDF risk 3, those with LOPS or mild PAD followed by a history of a foot ulcer or minor lower-extremity amputation would likely benefit from the use of special therapeutic footwear. Therefore, despite the second or third class of risk of DFU according to IWGDF classification, a person with diabetes and LOPS or mild PAD would more likely benefit from the use of special therapeutic footwear as long as excessive mechanical stress occurs at the plantar and dorsal surfaces of feet, which may become the main reason for a potential foot ulcer.

Our study has several strengths: (1) in the present meta-analysis, the updated pooled results regarding the efficacy of special therapeutic footwear in preventing foot ulcers were from data from RCTs, which would contribute to producing more convincing evidence. Based on the definition of special therapeutic footwear in the latest IWGDF Practical Guidelines (2019), we collected data from all eligible RCTs on special footwear and obtained powerful evidence that further supported the recommendation on special footwear in the aforementioned guidelines. (2) In the overall analysis of the main outcome, we selected the random-effect model and the Hartung-Knapp-Sidik-Jonkman method, which could reduce type I error and generate more robust results, particularly in the presence of substantial heterogeneity and a limited number of enrolled studies [43]. (3) Based on the data from all relevant RCTs, we first found that longer intervention time period worsened the efficacy of special therapeutic footwear in preventing diabetes-related foot ulcers, which suggested that more attention should be given to the relationship between patients' compliance with special therapeutic footwear or special therapeutic footwear durability and the effect of therapeutic footwear.

However, our findings should be interpreted cautiously due to some limitations. First, some included trials lacked a rigorous approach and complete reporting, such as sample size estimates, allocation concealment, blinding of assessors, or drop-out rates. Thus, the quality of these individual trials was variable and usually unclear, which might result in a high risk of bias in the current meta-analysis. Second, the diverse materials and design features of special therapeutic footwear as well as the different intervention times of the included trials might contribute to significant heterogeneity in our study. Third, reductions in peak pressure and footwear adherence are important factors that have the potential to significantly impact whether special therapeutic footwear produces improvement in plantar foot ulcer occurrence or recurrence. However, in this study, we were unable to perform statistical pooling for these key parameters because most of the included studies did not collect the relevant data or report them. Fourth, the severity of neuropathy, deformities, vascular status, and history of amputation may also influence DFU occurrence or recurrence. We extracted data, such as insensitivity to monofilament, VPT, foot deformities, peripheral artery disease, and a history of amputation. However, we were unable to obtain the respective incidence of DFU occurrence or recurrence among patients with different severities of neuropathy, foot deformities, peripheral artery disease, or a history of amputation. Thus, we were also unable to explore the influence of these potential factors on DFU occurrence or recurrence.

## 5. Conclusion

In conclusion, our analyses provide robust evidence that special therapeutic footwear with offloading properties significantly reduces the incidence of DFU. However, the effect may decrease gradually over time. Despite undefined reasons, the optimal utility time and renewal frequency of special therapeutic footwear should be considered.

## Figures and Tables

**(a):** 

Author/year	Country	Prevention target	Group	Number of participants	Age (years)	Male (%)	History of foot ulcer (%)	Intervention time (months)	HbA1c (%)	VPT (V) at baseline	BMI (kg/m^2^)	Duration of diabetes (years)	Incidence of foot ulcers
Bus et al., [6]	Netherlands	Secondary prevention	Intervention	85	62.6 ± 10.2	82	100	18	7.5 ± 1.4	50.0 ± 11.1	30.9 ± 6.4	19.9 ± 15.1	33
			Control	86	63.9 ± 10.1	83	100	18	7.6 ± 1.5	50.0 ± 9.0	30.4 ± 4.9	14.7 ± 11.2	38

Lavery et al., [27]	USA	Primary and secondary prevention	Intervention	149	69.4 ± 10.0	69	27.5	18	NR	29.8 ± 16.1	NR	13.0 ± 8.7	3
			Control	150	71.5 ± 7.9	67	25.3	18	NR	29.0 ± 15.1	NR	12.0 ± 4.9	10

Reiber et al., [28] ∗	USA	Secondary prevention	Intervention 1	121	61.0 ± 10.1	78	100	24	NR	NR	33.0 ± 6.8	NR	18
			Intervention 2	119	62.0 ± 10.1	77	100	24	NR	NR	32.0 ± 6.9	NR	17
			Control	160	63.0 ± 10.0	77	100	24	NR	NR	33.0 ± 7.2	NR	27

Rizzo et al., [29]∗∗	Italy	Primary and secondary prevention	Intervention	148	68.1 ± 14.1	68	NR	12	8.6 ± 1.4	26.1 ± 5.2	68.1 ± 14.1	17.4 ± 10.9	17
			Control	150	66.2 ± 9.4	66	NR	12	8.7 ± 1.1	27.6 ± 6.1	66.2 ± 9.4	18.1 ± 12.1	58

Scire et al., [30]	Italy	Primary prevention	Intervention	89	58.2 ± 17.1	NR	0	3	8.2 ± 1.7	37.4 ± 10.2	58.2 ± 17.1	15.2 ± 8.9	1
			Control	78	54.9 ± 18.2	NR	0	3	7.9 ± 0.9	34.1 ± 9.9	54.9 ± 18.2	16.4 ± 9.4	12

Uccioli et al., [31]	Italy	Secondary prevention	Intervention	33	59.6 ± 11.0	61	100	12	NR	33.0 ± 9.0	NR	16.8 ± 12.7	9
			Control	36	60.2 ± 8.2	64	100	12	NR	31.0 ± 12.0	NR	17.5 ± 8.0	21

Cisneros et al., [32]	Brazil	Primary and secondary prevention	Intervention	30	64.4 ± 9.2	63	26.7	24	NR	NR	NR	14.0 ± 10.0	8
			Control	23	59.8 ± 9.0	36	34.8	24	NR	NR	NR	15.0 ± 10.5	8

Ulbrecht et al., [33]	USA	Secondary prevention	Intervention	66	60.5 ± 10.1	76	100	15	NR	NR	32.3 ± 7.1	NR	6
			Control	64	58.5 ± 10.7	81	100	15	NR	NR	31.4 ± 5.5	NR	16

**(b):** 

Author/year	Country	Group	Number of participants	Adherence (%)	Insensate to monofilament (%)	Peripheral artery disease (%)	Foot deformity (%)	History of minor amputation (%)
Bus et al., [6]	Netherlands	Intervention	85	41.2	94.1	28.8	95.3	0
		Control	86	51.2	91.9	37.5	97.7	0

Lavery et al., [27]	USA	Intervention	149	4 h/d: 15.5 4-8 h/d: 52.0 8-12 h/d: 25.7 12-16 h/d: 6.8	NR	0	NR	12.1
		Control	150	4 h/d: 10.6 4-8 h/d: 55.0 8-12 h/d: 30.5 12-16 h/d: 3.9	NR	0	NR	8.7

Reiber et al., [28]∗	USA	Intervention 1	121	83.0	59	NR	36	0
		Intervention 2	119	86.0	66	NR	22	0
		Control	160	NR	52	NR	35	0

Rizzo et al., [29]∗∗	Italy	Intervention	148	NR	NR	NR	NR	NR
		Control	150	NR	NR	NR	NR	NR

Scire et al., [30]	Italy	Intervention	89	NR	NR	0	6	NR
		Control	78	NR	NR	0	8	NR

Uccioli et al., [31]	Italy	Intervention	33	100	NR	NR	NR	0
		Control	36	NR	NR	NR	NR	0

Cisneros et al., [32]	Brazil	Intervention	30	≤6 h/d: 34.5 >6 h/d: 37.9 Not daily: 27.6	NR	NR	50.0	NR
		Control	23	NR	NR	NR	30.4	NR

Ulbrecht et al., [33]	USA	Intervention	66	NR	NR	NR	NR	31.8
		Control	64	NR	NR	NR	NR	37.5

Data are shown as numbers, mean ± SD or %. NR, not reported; BMI, body mass index; VPT, vibration perception threshold; HbA1c, glycosylated hemoglobin. ∗ This study had two intervention groups (intervention 1: custom cork-insert group; intervention 2: polyurethane insert group). For the meta-analyses the intervention 1 group and intervention 2 were incorporated into a single intervention group to compare with control group. ∗∗ This study reported only the overall proportions of patients with foot deformities (46%), history of foot ulcer (20%), and history of minor amputation (25%) among all participants.

**Table 2:** Risk of bias of included studies.

Author/year	Adequate randomization sequence generation	Adequate blinding of participants	Adequate blinding of assessors	Adequate allocation concealment	Free from incomplete outcome data	Free from selective reporting	Sample size estimate	Total risk of bias
Bus et al., [6]	Definitely yes	Definitely yes	Definitely yes	Definitely yes	Definitely yes	Definitely yes	Probably yes	Low risk
Lavery et al., [27]	Probably yes	Definitely no	Definitely yes	Probably yes	Definitely yes	Definitely yes	Definitely yes	High risk
Reiber et al., [28]	Definitely yes	Probably yes	Definitely yes	Probably yes	Definitely yes	Definitely yes	Definitely yes	Low risk
Rizzo et al., [29]	Definitely yes	Probably yes	Probably yes	Probably yes	Definitely yes	Definitely yes	Definitely no	High risk
Scire et al., [30]	Definitely yes	Probably yes	Definitely yes	Probably yes	Definitely yes	Definitely yes	Definitely no	High risk
Uccioli et al., [31]	Probably yes	Probably yes	Probably yes	Probably yes	Definitely yes	Definitely yes	Definitely no	High risk
Cisneros et al., [32]	Definitely yes	Definitely yes	Probably yes	Probably yes	Definitely yes	Definitely yes	Probably yes	Low risk
Ulbrecht et al., [33]	Definitely yes	Definitely no	Definitely yes	Definitely yes	Definitely yes	Definitely yes	Definitely yes	High risk

## Data Availability

The data supporting this meta-analysis are from previously reported studies and datasets, which have been cited.

## Supplementary Materials

Supplementary 1 Appendix 1: Search strategy.

Supplementary 2 Appendix 2: PRISMA checklist.

Supplementary 3 Appendix 3: Subgroup analysis for patients with a history of foot ulceration.
