# Supplementary material for: Effects of Special Therapeutic Footwear on the Prevention of Diabetic Foot Ulcers: A Systematic Review and Meta-Analysis of Randomized Controlled Trials
Source: J Diabetes Res. 2022 Sep 26;2022:9742665. doi: 10.1155/2022/9742665 (PMC9530919; doi:10.1155/2022/9742665)
Supplement: Supplementary 1 — Appendix 1: Search strategy. [file 9742665.f1.docx]

Appendix 1：PRISMA checklist (This meta-analysis was followed the PRISMA statement, and PRISMA checklist was uploaded as supplementary materials.)

| **Section/Topic** | **#** | **Checklist Item** | **Reported on Page #** |
| --- | --- | --- | --- |
| **TITLE** |  |  |  |
| Title | 1 | Identify the report as a systematic review, meta-analysis, or both. | Page 1 |
| **ABSTRACT** |  |  |  |
| Structured summary |  | Provide a structured summary including, as applicable: background; objectives; data sources; study eligibility criteria, participants, and interventions; study appraisal and synthesis methods; results; limitations; conclusions and implications of key findings; systematic review registration number. | Page 2 to 3(line 29-54) |
| **INTRODUCTION** |  |  |  |
| Rationale | 3 | Describe the rationale for the review in the context of what is already known. | Page 4 to 5(line 61-88) |
| Objectives | 4 | Provide an explicit statement of questions being addressed with reference to participants, interventions, comparisons, outcomes, and study design (PICOS). | Page 5(line 88-93) |
| **METHODS** |  |  |  |
| Protocol and registration | 5 | Indicate if a review protocol exists, if and where it can be accessed (e.g., Web address), and, if available, provide registration information including registration number | In fact, we are now conducting a two arms, parallel-group, open-label randomized controlled trial in several diabetic foot centers of China to examine the effect of an optimally designed therapeutic footwear on preventing ulcer recurrence in patients with a history of diabetic foot ulcers (DFU). The Trial registration is Chinese Clinical Trial Registry ChiCTR1900025538 and the protocol have published in the journal, “Trials”*[Trials. 2021;22:151]*. During the period of designing the RCT, we searched and read a lot of literature and then intended to complete a review based on the available clinical evidence. Because the present systematic and meta-analysis was considered by us as a part of the whole project, we did not register our systematic review and meta-analysis in PROSPERO database. |
| Eligibility criteria | 6 | Specify study characteristics (e.g., PICOS, length of follow-up) and report characteristics (e.g., years considered, language, publication status) used as criteria for eligibility, giving rationale. | Page 6 (line109-117) |
| Information sources | 7 | Describe all information sources (e.g., databases with dates of coverage, contact with study authors to identify additional studies) in the search and date last searched. | Page 5 to 6(line100-106) |
| Search | 8 | Present full electronic search strategy for at least one database, including any limits used, such that it could be repeated. | Supplementary Materials (Appendix 2) |
| Study selection | 9 | State the process for selecting studies (i.e., screening, eligibility, included in systematic review, and, if applicable, included in the meta-analysis). | Page 5-7(line 99-132) |
| Data collection process | 10 | Describe method of data extraction from reports (e.g., piloted forms, independently, in duplicate) and any processes for obtaining and confirming data from investigators. | Page 7(line123-127) |
| Data items | 11 | List and define all variables for which data were sought (e.g., PICOS, funding sources) and any assumptions and simplifications made. | Page 7(line128-132) |
| Risk of bias in individual  studies | 12 | Describe methods used for assessing risk of bias of individual studies (including specification of whether this was done at the study or outcome level), and how this information is to be used in any data synthesis. | Page 7 (line 134-143) |
| Summary measures | 13 | State the principal summary measures (e.g., risk ratio, difference in means). | Page 8(line 148-150) |
| Synthesis of results | 14 | Describe the methods of handling data and combining results of studies, if done, including measures of consistency (e.g., I2) for each meta-analysis. | Page 8(line 150-164) |
| Risk of bias across studies | 15 | Specify any assessment of risk of bias that may affect the cumulative evidence (e.g., publication bias, selective reporting within studies). | Page 9(line 171-172) |
| Additional analyses | 16 | Describe methods of additional analyses (e.g., sensitivity or subgroup analyses, meta-regression), if done, indicating which were pre-specified. | Page 8 (line 165-170)  We added the methods of sensitivity analyses (including searching for outliers and influence analysis) and meta-regression analyses. |
| **RESULTS** |  |  |  |
| Study selection | 17 | Give numbers of studies screened, assessed for eligibility, and included in the review, with reasons for exclusions at each stage, ideally with a flow diagram. | Figure 1 |
| Study characteristics | 18 | For each study, present characteristics for which data were extracted (e.g., study size, PICOS, follow-up period) and provide the citations. | Page 9(line 180-189); Table 1 |
| Risk of bias within studies | 19 | Present data on risk of bias of each study and, if available, any outcome-level assessment (see Item 12). | Page 10 (line 192-198); Table 2 |
| Results of individual  studies | 20 | For all outcomes considered (benefits or harms), present, for each study: (a) simple summary data for each intervention group and (b) effect estimates and confidence intervals, ideally with a forest plot. | Figure 2 |
| Synthesis of results | 21 | Present results of each meta-analysis done, including confidence intervals and measures of consistency. | Page 10 (line 200-204); Figure 2 |
| Risk of bias across studies | 22 | Present results of any assessment of risk of bias across studies (see Item 15). | Page 11(line 215-219) |
| Additional analysis | 23 | Give results of additional analyses, if done (e.g., sensitivity or subgroup analyses, meta-regression [see Item 16]). | Page 11(line 206-213); Figure 3; Appendix 3(Figure 5) |
| **DISCUSSION** |  |  |  |
| Summary of evidence | 24 | Summarize the main findings including the strength of evidence for each main outcome; consider their relevance to key groups (e.g., health care providers, users, and policy makers). | Page 11 to 17 (line 224-334) |
| Limitations | 25 | Discuss limitations at study and outcome level (e.g., risk of bias), and at review level (e.g., incomplete retrieval of identified research, reporting bias). | Page 17 to 18 (line 335-354) |
| Conclusions | 26 | Provide a general interpretation of the results in the context of other evidence, and implications for future research. | Page 18 (line 356-361) |
| **FUNDING** |  |  |  |
| Funding | 27 | Describe sources of funding for the systematic review and other support (e.g., supply of data); role of funders for the systematic review. | Page 19 (line 373-378) |
