# Supplementary material for: Effects of Special Therapeutic Footwear on the Prevention of Diabetic Foot Ulcers: A Systematic Review and Meta-Analysis of Randomized Controlled Trials
Source: J Diabetes Res. 2022 Sep 26;2022:9742665. doi: 10.1155/2022/9742665 (PMC9530919; doi:10.1155/2022/9742665)
Supplement: Supplementary 2 — Appendix 2: PRISMA checklist. [file 9742665.f2.docx]

**Appendix 2：**

**1.1** **search strategy for Embase (OVID)**

exp Diabetic Foot/

(diabet* adj3 (foot or feet)).ti,ab,kw.

exp Foot Ulcer/

(diabet* adj3 ulcer*).ti,ab,kw.

(diabet* adj3 wound*).ti,ab,kw.

or/1-5

exp walking aid/

pressure relie*.ti,ab,kw.

pressure reduc*.ti,ab,kw.

(pressure adj (distribut* or redistribut*)).ti,ab,kw.

(alternat* adj pressure).ti,ab,kw.

constant pressure.ti,ab,kw.

exp Walkers/

off?load*.ti,ab,kw.

walker*.ti,ab,kw.

exp orthosis/

(orthoses or orthosis).ti,ab,kw.

Orthotic*.ti,ab,kw.

exp shoe/

exp orthopedic shoe/

(shoe* or boot* or footwear).ti,ab,kw.

((therapeutic or bespoke or semibespoke or manufactured or custom*) adj3 (footwear or shoe*)).ti,ab,kw.

(sandal or sandals).ti,ab,kw.

(non-weightbearing or nonweightbearing).ti,ab,kw.

nonweight bearing.ti,ab,kw.

non-weight bearing.ti,ab,kw.

insole*.ti,ab,kw.

foot insert*.ti,ab,kw.

cushion*.ti,ab,kw.

(heel adj elevat*).ti,ab,kw.

(elevat* adj device*).ti,ab,kw.

(foot adj elevat*).ti,ab,kw.

(foot adj lift*).ti,ab,kw.

or/7-33

Clinical trial/

Randomized controlled trial/

Randomization/

Single blind procedure/

Double blind procedure/

Crossover procedure/

Randomi?ed controlled trial*.ti,ab,kw.

Rct.ti,ab,kw.

Random allocation.ti,ab,kw.

Randomly allocated.ti,ab,kw.

Allocated randomly.ti,ab,kw.

(allocated adj2 random).ti,ab,kw.

Single blind*.ti,ab,kw.

Double blind*.ti,ab,kw.

((treble or triple) adj blind*).ti,ab,kw.

or/35-49

case study/

case report.ti,ab,kw.

abstract report/

letter/

or/51-54

50 not 55

Clinical study/

Case control study/

Family study/

Longitudinal study/

Retrospective study/

Prospective study/

Cohort analysis/

(Cohort adj (study or studies)).ti,ab,kw.

(Case control adj (study or studies)).ti,ab,kw.

(follow up adj (study or studies)).ti,ab,kw.

(observational adj (study or studies)).ti,ab,kw.

(epidemiologic* adj (study or studies)).ti,ab,kw.

or/57-68

6 and 34 and 69

**1.2 search strategy for Medline (OVID)**

exp Diabetic Foot/

(diabet* adj3 (foot or feet)).tw.

exp Foot Ulcer/

(diabet* adj3 ulcer*).tw.

(diabet* adj3 wound*).tw.

or/1-5

exp Walking/

exp Walkers/

pressure relie*.tw.

pressure reduc*.tw.

(pressure adj (distribut* or redistribut*)).tw.

(alternat* adj pressure).tw.

constant pressure.tw.

off?load*.tw.

walker*.tw.

exp Orthotic Devices/

(orthoses or orthosis).tw.

Orthotic*.tw.

exp shoe/

(shoe* or boot* or footwear).tw.

((therapeutic or bespoke or semibespoke or manufactured or custom*) adj3 (footwear or shoe*)).tw.

(sandal or sandals).tw.

(non-weightbearing or nonweightbearing).tw.

nonweight bearing.tw.

non-weight bearing.tw.

insole*.tw.

foot insert*.tw.

cushion*.tw.

(heel adj elevat*).tw.

(elevat* adj device*).tw.

(foot adj elevat*).tw.

(foot adj lift*).tw.

or/7-32

clinical trial/

clinical trial, phase i.pt.

clinical trial, phase ii.pt.

clinical trial, phase iii.pt.

clinical trial, phase iv.pt.

controlled clinical trial.pt.

randomized controlled trial.pt.

multicenter study.pt.

clinical trial.pt.

exp Clinical Trial/

or/34-43

(clinical adj trial*).tw.

((singl* or doubl* or treb* or tripl*) adj (blind* or mask*)).tw.

randomly allocated.tw.

(allocated adj2 random*).tw.

exp Randomized Controlled Trials as Topic/

or/45-49

44 or 50

Case study/

case report.tw.

letter/

or/52-54

51 not 55

exp Cohort Studies/

Epidemiology/

Case control.tw.

(cohort adj (study or studies)).tw.

Cohort analy*.tw.

(Follow up adj (study or studies)).tw.

(observational adj (study or studies)).tw.

Longitudinal.tw.

Retrospective.tw.

or/57-65

56 or 66

exp animals/ not humans.sh.

67 not 68

6 and 33 and 69

**1.3 search strategy for EMB databases (OVID)**

exp Diabetic Foot/

(diabet* adj3 (foot or feet)).tw.

exp Foot Ulcer/

(diabet* adj3 ulcer*).tw.

(diabet* adj3 wound*).tw.

or/1-5

exp Walking/

exp Walkers/

pressure relie*.tw.

pressure reduc*.tw.

(pressure adj (distribut* or redistribut*)).tw.

(alternat* adj pressure).tw.

constant pressure.tw.

off?load*.tw.

walker*.tw.

exp Orthotic Devices/

(orthoses or orthosis).tw.

Orthotic*.tw.

exp shoe/

(shoe* or boot* or footwear).tw.

((therapeutic or bespoke or semibespoke or manufactured or custom*) adj3 (footwear or shoe*)).tw.

(sandal or sandals).tw.

(non-weightbearing or nonweightbearing).tw.

nonweight bearing.tw.

non-weight bearing.tw.

insole*.tw.

foot insert*.tw.

cushion*.tw.

(heel adj elevat*).tw.

(elevat* adj device*).tw.

(foot adj elevat*).tw.

(foot adj lift*).tw.

or/7-32

clinical trial/

clinical trial, phase i.pt.

clinical trial, phase ii.pt.

clinical trial, phase iii.pt.

clinical trial, phase iv.pt.

controlled clinical trial.pt.

randomized controlled trial.pt.

multicenter study.pt.

clinical trial.pt.

exp Clinical Trial/

or/34-43

(clinical adj trial*).tw.

((singl* or doubl* or treb* or tripl*) adj (blind* or mask*)).tw.

randomly allocated.tw.

(allocated adj2 random*).tw.

exp Randomized Controlled Trials as Topic/

or/45-49

44 or 50

exp Cohort Studies/

Epidemiology/

Case control.tw.

(cohort adj (study or studies)).tw.

Cohort analy*.tw.

(Follow up adj (study or studies)).tw.

(observational adj (study or studies)).tw.

Longitudinal.tw.

Retrospective.tw.

or/52-60

51 or 61

6 and 33 and 62
