# Supplementary material for: Effects of Special Therapeutic Footwear on the Prevention of Diabetic Foot Ulcers: A Systematic Review and Meta-Analysis of Randomized Controlled Trials
Source: J Diabetes Res. 2022 Sep 26;2022:9742665. doi: 10.1155/2022/9742665 (PMC9530919; doi:10.1155/2022/9742665)
Supplement: Supplementary 3 — Appendix 3: Subgroup analysis for patients with a history of foot ulceration. [file 9742665.f3.pdf]

Appendix 3: Subgroup analysis for patients with a history of foot ulceration.

Study	Experimental Events	Experimental Total	Control Events	Control Total	Risk Ratio	RR	95%−CI	Weight
reiber2002	35	240	27	160		0.86	[0.55; 1.37]	28.7%
uccioli1995	9	33	21	36		0.47	[0.25; 0.87]	22.7%
bus2013	33	85	38	86		0.88	[0.61; 1.26]	33.0%
ulbrecht2014	6	66	16	64		0.36	[0.15; 0.87]	15.6%
**Random effects model**		**424**		**346**		**0.66**	**[0.34; 1.28]**	**100.0%**

Heterogeneity: $I^2 = 50\%$, $\tau^2 = 0.1140$, $p = 0.11$

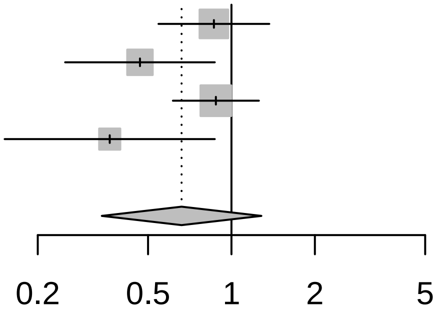

Figure 5. Effect of special therapeutic footwear in reducing the recurrence of diabetic foot ulcers among patients with a history of foot ulceration.
